# Redesigning Carbon–Carbon Backbone Polymers for Biodegradability–Compostability at the End-of-Life Stage

**DOI:** 10.3390/molecules28093832

**Published:** 2023-04-30

**Authors:** Neha Mulchandani, Ramani Narayan

**Affiliations:** Department of Chemical Engineering and Materials Science, Michigan State University, East Lansing, MI 48824, USA

**Keywords:** carbon–carbon backbone, biodegradable–compostable, polymers, end-of-life, biobased, carbon footprint

## Abstract

Carbon–carbon backbone polymers are non-biodegradable, persistent plastics that have accumulated on land and oceans due to human activities. They degrade and fragment into microplastics and smaller particle sizes but do not biodegrade at an acceptable and practical rate. Their continual buildup in the natural environment precipitates serious detrimental impacts on human health and the environment, as extensively documented in the literature and media. Nearly 77% of global plastics produced are carbon–carbon backbone polymers. More importantly, 90% of packaging plastics (153.8 million metric tons) are non-biodegradable, persistent carbon–carbon backbone polymers. The recycling rate of these non-durable packaging plastics ranges from 0 to 4%. Re-designing carbon–carbon backbone polymers to labile ester backbone biodegradable–compostable polymers and treating them along with biodegradable organic waste (such as food, paper, and organic wastes) in managed industrial composting is environmentally responsible. Diverting 1 million metric tons of biodegradable organic wastes in MSW bound for landfills and open dumps to industrial composting results in 0.95 million metric tons CO_2_ equivalents of GHG emissions reduction. This perspective paper discusses strategies and rationales regarding the redesign of carbon–carbon backbone polymer molecules. It describes the carbon footprint reductions achievable by replacing petro-fossil carbon with plant biomass carbon. Biodegradability and compostability are frequently used but misunderstood and misused terms, leading to misleading claims in the marketplace. This paper presents the fundamentals of biodegradability and compostability of plastics and the requirements to be met according to ASTM/ISO international standards.

## 1. Introduction

The production of plastics has experienced enormous growth since the 1950s, surpassing most other classes of materials. The global production of plastics accounted for 9.2 billion tons in 2017, which is projected to rise to 34 billion tons by 2050 [1]. The most commonly used plastics are polyolefins, which are derived from fossil hydrocarbons [2]. Nearly 77% of the plastics produced globally consist of polymers possessing a carbon–carbon backbone architecture, providing them with durability that allows them to resist degradation in the environment [3]. In turn, this results in long-lasting materials that prevail in the environment for decades or longer unless destroyed by a thermal treatment such as incineration [4]. The largest plastic demand according to application is that of packaging, which has an ephemeral usage life prior to becoming waste. Polymers such as polyethylene (PE), polypropylene (PP), and polystyrene (PS) are carbon–carbon backbone polymers that account for ~77% of the polymers used in packaging applications. It has been determined that an estimated 12 billion tons of plastic waste will have accumulated on the earth by 2050 if the rate of plastic production, utilization, and waste management continues at the current pace [5]. The growing volumes of plastic production have raised serious concerns regarding their impact on ecosystems and human health. However, little attention has been paid to their impact on climate change, which often occurs in the form of greenhouse gas (GHG) emissions [6,7]. Furthermore, it is known that carbon–carbon backbone polymers contribute to significantly greater amounts of greenhouse gas (GHG) emissions [8,9]; accordingly, the single-use application of these polymers is now being prohibited [10]. The GHG emissions pertaining to carbon–carbon backbone polymers amounted to 1.7 GT CO_2_e (Gigatonnes of CO_2_ equivalent) in 2015, which is expected to increase to 6.5 GT CO_2_e by 2050 if the production of carbon–carbon backbone polymers continues on its current trajectory [11]. The strategies for mitigating the existing climate issues include reducing the accumulation of plastics by lowering demand, increasing the number of avenues for recycling plastics, or replacing carbon–carbon backbone polymers with sustainable end-of-life (EoL) solutions [12,13]. The recycling streams of plastics are often mixed or contaminated [14], necessitating excessive sorting and cleaning and thus making their separation difficult [15]. To address the issue of plastic waste, especially non-durable, single-use packaging and products, it is important to chemically redesign carbon–carbon backbone polymers such that the usage of renewable, biobased carbon is included in their production [16], incorporating labile oxygen linkages and developing methods for their responsible end-of-life management [17]. Redesigning polymers by incorporating an ester backbone can introduce the property of biodegradability–compostability into plastics, which can be diverted to managed industrial composting systems, thereby reducing the amount of plastics entering natural environments such as oceans and land [18]. Unfortunately, there is a great deal of misunderstanding and misleading product claims regarding biodegradability and compostability in the marketplace. This is especially true of claims concerning the induction of biodegradability in carbon–carbon backbone polymers through the use of oxo or organic additives. The requirements for assessing and reporting plastic biodegradation have been published [19]. Further, the outbreak of the COVID-19 pandemic led to an increased demand for single-use plastic products, particularly marks, visors, protective suits, packaging for goods sold online, and containers for the storage and transportation of medical waste as well as food and meals. The extensive use of plastics, especially in a single-use form as disposable packaging and other products, has posed serious concerns in connection with their carbon footprint and end-of-life characteristics [20]. 

This perspective paper discusses the redesigning of carbon–carbon backbone polymers such that they can be biodegradable–compostable at their end of life, with the aim of providing a value proposition to reduce carbon footprints and provide responsible end-of-life properties. The science of the biodegradability and compostability of polymers and the use of biobased carbon is reviewed. Since biodegradability and compostability are frequently used and misunderstood terms due to the misleading claims propagated in the marketplace, the present paper presents the fundaments of the biodegradability and compostability of plastics and the requirements to be met according to ASTM/ISO standards in specific environments.

## 2. Bioplastics for a Managed End of Life

Bioplastics [21] comprise two categories: biobased plastics and biodegradable–compostable plastics [22]. Biobased plastics are entirely connected to the origin of the carbon in the plastic, while biodegradable–compostable plastics have been termed in reference to their end-of-life characteristics in a specific environment and at a specific time, such as industrial composting [23]. It is crucial to recognize that a biodegradable–compostable plastic may be derived from fossil-based resources and that a biobased plastic may not be biodegradable at its end of life, or vice versa [24]. Additionally, biodegradability is not only an inherent property of a material but also depends on the properties of the receiving environment [25]. Therefore, the biodegradability of certified plastics must be determined in the receiving environment for which the plastic is certified as biodegradable. For instance, a plastic that is certified as industrially compostable cannot be investigated with respect to its insufficient biodegradation in marine environments [26]. Therefore, it becomes crucial to recognize the key properties of the plastics as well as the receiving environment while assessing the biodegradation of plastics [27].

**Figure 1:**
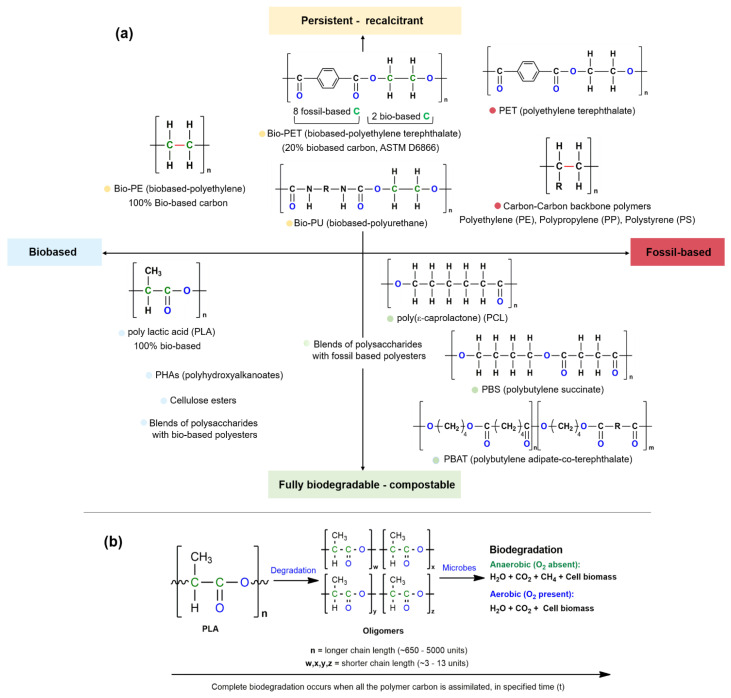
(**a**) Polymers depicted according to their feedstock and biodegradability–compostability; (**b**) Complete biodegradation process of biodegradable-compostable polymers. Reproduced with permission from REF [17].

Relevant polymers have been characterized according to their carbon feedstock and biodegradability–compostability, as shown in Figure 1a. The conventional carbon–carbon backbone polymers, such as polypropylene (PP), polyethylene (PE), and polystyrene (PS), are resistant to biodegradation due to their carbon–carbon bond strength. Their non-biodegradability leads to their persistence, thereby creating challenges with respect to their waste management and accumulation in oceans and on land. The fragmentation of carbon–carbon backbone polymers can be achieved via photochemical degradation; however, complete oxidation occurs over decades or centuries, which is a relatively long time-scale. On the contrary, the chemical linkages in a biodegradable polymer’s backbone are susceptible to biotic or abiotic reactions, which convert the polymer into smaller fragments or oligomeric species that are completely metabolized by microorganisms (Figure 1b) in a defined system, such as agricultural soil, an anaerobic digester, industrial compost, or wastewater, within a specified time. The specified environmental conditions further allow for the complete metabolization of plastic on a practical timescale that allows for the mineralization of CO_2_. The existing polymers that are derived from natural sources (bio-based) and are biodegradable–compostable at their end-of-life stage include polylactic acid (PLA) [28], polyhydroxyalkanoates (PHA) [29], cellulose esters [30], and polysaccharides [18]. The rate of biodegradation of cellulose is dependent on temperature, which demonstrates the effect of environment on polymer degradation. PLA is a biobased polymer [31] that complies with the criteria of industrial compostability; however, it is not biodegradable under normal environmental conditions. Therefore, designing polymers that can degrade in a natural system becomes challenging, which further necessitates the requirements of specified composting systems for the biodegradation of polymers [32]. Additionally, the differences in the microbial populations in a test system as compared to a natural environment necessitate the use of standard test methods for determining the biodegradation of plastics [33]. Some of the commercially available biodegradable–compostable polymers are shown in Table 1.

### 2.1. Biodegradation of Polymers

**Figure 2:**
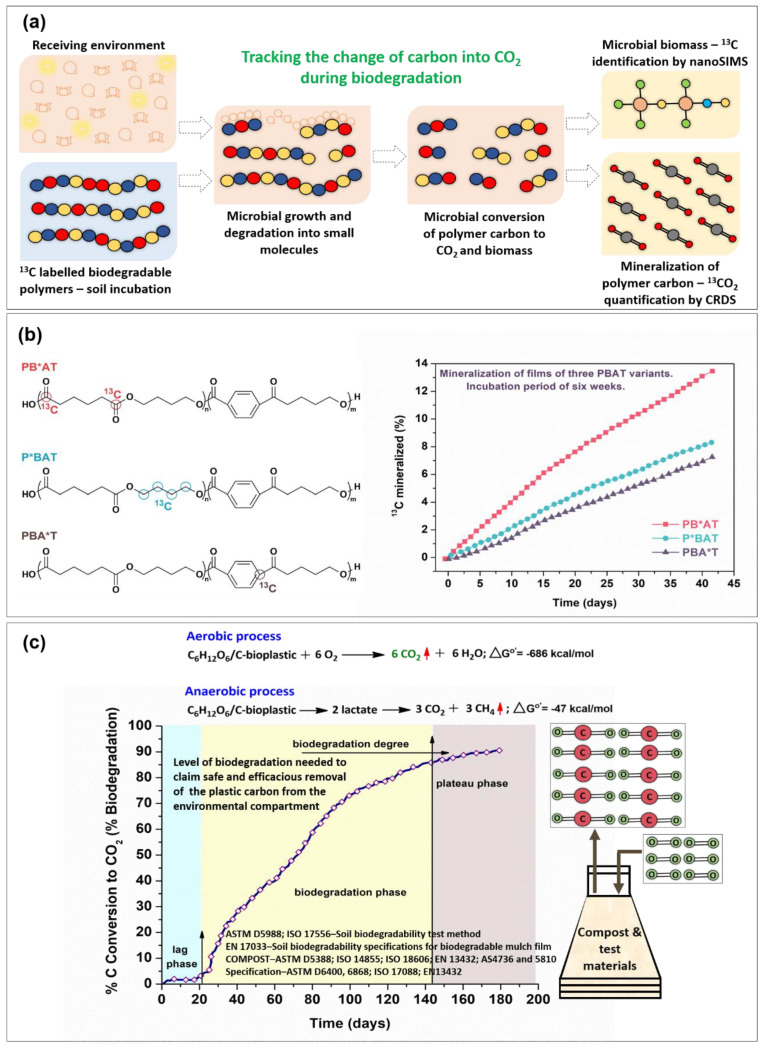
(**a**) Biodegradation of plastics based on carbon-isotope-labelling approach; (**b**) mineralization of PBAT variants differing in monomer units; and (**c**) respirometric graphical analysis according to the application of standards. Parts (**a**,**b**) were reproduced with permission of AAAS from [59]. © The authors, some rights reserved; exclusive licensee AAAS. Distributed under a CC BY-NC 4.0 License (http://creativecommons.org/licenses/by-nc/4.0/, Accessed on 17 January 2023). Part (**c**) was reproduced with permission from [58].

The biodegradation of polymers is defined as the microbial conversion of nearly all the polymeric carbon (90%+) to CO_2_, which releases energy that is harnessed by microbes for their life processes. This highly specialized cellular phenomenon takes place inside the cell and requires the participation of three basic metabolically interrelated processes: the TCA (tricarboxylic acid) cycle, electron transport, and oxidative phosphorylation. Therefore, the quantitative measurement of the conversion of polymeric carbon to CO_2_ (or CO_2_ and methane in an anaerobic process) is essential for assessing the biodegradation of plastics. Biodegradation is a function of the physicochemical characteristics of a polymer and the biochemical nature of the corresponding disposal system. Such microbial respirometric data are absent for some products purported to be biodegradable, such as “oxo-biodegradable” plastics. These oxo-biodegradable formulations essentially contain specific additives added to carbon–carbon backbone polymers such as polyethylenes, polypropylenes, and polystyrenes. It is claimed that these additive formulated persistent hydrocarbon plastics degrade and, ultimately, completely biodegrade. The data provided supporting these claims involve weight loss, microbial film formation, carbonyl index values, and other physical–chemical characterizations. However, no respirometric data (using ISO/ASTM international test methods) showing 90%+ polymer conversion to CO_2_ via microbial metabolism in an acceptable and practical time frame have been documented for oxo-biodegradable formulations. Detailed guidance for respirometric analyses in specific environments and specifications can be obtained from the ASTM and ISO standards. Figure 2c shows a respirometric graphical analysis that was performed according to the application of the standards [58]. 

Various methods have been proposed for the tracking of a polymer’s carbon in CO_2_ and microbial biomass, for which the approach based on carbon-isotope-labelled plastics (^13^C labelling) [59] is the most suitable for tracking a plastic’s carbon conversion to CO_2_ and microbial biomass. A schematic representation of plastic biodegradation is shown in Figure 2a, where microbial growth occurs on the ^13^C-labelled plastics in the receiving environment, leading to the polymer chain’s scission and the conversion of the carbon contained in the plastic to CO_2_ and biomass. Nanoscale secondary ion mass spectroscopy (nanoSIMS) allows for the identification of ^13^C-labelled carbon in the biomass, whereas the mineralization of polymeric carbon into CO_2_ was reported via Cavity Ring-Down Spectroscopy (CRDS). Figure 2b shows the biodegradability of PBAT (polybutylene adipate-co-terephthalate), where the three variants of PBAT differing in monomer units were labelled with the ^13^C-isotope. The mineralization of PBAT variants was observed over the course of six weeks, and the formation of ^13^CO_2_ was monitored using CRDS. 

Experimental findings, including the visual disappearance of plastic, mass loss, reduction in the average polymer chain length, loss of mechanical properties, microbial growth, etc., have been referred to by the researchers so that they could claim that the developed plastics are biodegradable. However, these investigations are not directly related to the conversion of the carbon in plastic into CO_2_ and, therefore, are not acceptable for determining the biodegradability of plastics. Experimental international standards have been issued for the strict assessment of the biodegradability of plastics in different environments including soil, compost, and marine environments, which are discussed below.

#### 2.1.1. Industrial Composting

Since compostability is an essential end-of-life property, standard protocols exist for testing the biodegradability of plastics in industrial composting [60] under controlled conditions [61,62]. The degree of biodegradability is ascertained by measuring the extent of conversion of >90% of a polymer’s carbon into CO_2_ within 180 days in a temperature-controlled (58 ± 2 °C), closed chamber relative to the total amount of polymeric carbon. The standard test further necessitates the disintegration of plastics over 84 days of composting, for which <10% of the plastic by dry-weight remains on a 2 mm sieve [61]. The microorganisms in the aerobic systems convert the polymeric carbon into CO_2_, thereby releasing energy for their life processes [63], whereas under anaerobic conditions, CO_2_, methane, and cellular biomass is formed [64].

#### 2.1.2. Home Composting

Home composting involves variable conditions, and the generated process heat can be dissipated quickly, resulting in lower temperatures. Thus, the rate of biodegradation is slower, highly variable, and less-effective compared to industrial composting. The U.S. EPA (United States Environmental Protection Agency) requires that the compost pile temperature is maintained at 55 °C (130 °F) for three days to kill pathogens and weeds, but such a temperature is not reached in home composting. The European standard for home compostability (PREN17427) requires 90% or greater conversion of polymer’s carbon to CO_2_ at 25+/−5 °C in a time frame of one year or less [65].

The successful use of compostable plastics in conjunction with food waste industrial composting has been extensively documented. However, there are studies that have raised questions requiring resolution [66,67]. More careful analysis is required with respect to the test protocols and methodology for home composting [68].

#### 2.1.3. Soil Biodegradation

A standard test method exists for determining the biodegradability of plastics in soil [69]. The specifications for biodegradable plastic mulch films suitable for horticultural applications are stipulated by the European Union [70]. Based on standard laboratory methods, >90% of the biodegradable mulch film’s polymeric carbon must be converted to CO_2_ within <24 months in the temperature range of 20–28 °C. However, the type of soil and the corresponding climatic conditions govern the degradation of plastics in soil, which is further affected by the temperature, microbial populations, and moisture content, which usually vary from soil to soil and according to the corresponding climatic conditions. Therefore, the biodegradation of plastics may differ in the field compared to the standard laboratory tests, with the field version usually being slower. This further necessitates in situ field testing at varying locations with different climates in order to complement the standard tests [71]. California recently passed a law—CA AB1201, which stipulates that mulch film must meet EN 17033:2018 entitled “Plastics-Biodegradable mulch films for use in agriculture and horticulture – Requirements and test methods” along with the ASTM standard certification for compostability (ASTM D6400 or ASTM D6868)—that allows for the commercial sale of agricultural mulch films (not intended to be removed from the field) labelled as ‘soil biodegradable’ [72]. 

#### 2.1.4. Marine Biodegradation

Marine litter has been recognized as a major threat to the environment. Currently, there are no standards that provide clear pass/fail criteria for the degradation of plastics in marine environments. However, the test methods that do not offer any pass/fail criteria, and are still in place, include ASTM D6691, D6692, D7473, OECD 306, and ISO 16621. The standardization efforts for the marine biodegradability of plastics are in progress and are currently at the ISO level. ISO 18830 and ISO 19679 are the standards for the determination of the aerobic biodegradation of non-floating plastic materials at the seawater/sediment interface. ISO 22404 is another standard for determining the biodegradation of non-floating materials in marine sediments. ISO 22403 includes requirements for inherent aerobic biodegradability. ISO 22766 involves a disintegration test of plastic materials in marine habitats under real field conditions [73]. These standards, however, are predominantly guidelines and do not clearly state condition requirements and timeframes. Further research and development is necessary to create standards for the marine biodegradation of plastics before introducing relevant products to the market [74].

From the above discussion, it can be discerned that the biodegradation of plastics is strongly dependent on system factors, thus reiterating that claims of certain plastics being biodegradable, non-biodegradable, or insufficiently biodegradable are relevant when discussed in the context of a receiving environment. For instance, PLA is a biobased polymer that is also certified as being industrially compostable; however, it does not biodegrade under natural environmental conditions.

#### 2.1.5. Microplastics

After their service life, plastics are sent to landfills or aquatic environments where they break down into smaller particles via weathering. The breakdown of plastics into the smaller particles (<5 μm) leads to the formation of microplastics [75], which constitute a diverse class of contaminants with complex compositions and varying shapes (in terms of fragments, spheres, and fibers). ASTM WK72349 delineates the test method for determining the particle and fiber size of microplastics [76]. Microplastics are prevalent in the global biosphere [77] and can be produced by the weathering of plastic products such as clothing, car tires, paints, electronic coatings, and so on. Moreover, they are knowingly added to routine products such as cosmetics and abrasive cleaners. The pervasiveness of microplastics [78] has precipitated serious impacts on wildlife and ecosystems [79] along with human health as they make their way through the human body via ingestion and inhalation. Such microplastics are taken up by various organs, thereby damaging cells [80] and, in turn, inducing inflammatory and immune reactions [81]. 

Biodegradable–compostable plastics also produce small particles through abrasion during their service life; however, these particles will biodegrade unlike the persistent microplastics that are produced using carbon–carbon backbone polymers. Biodegradable–compostable polymers are metabolized by microbes in most natural environments, which prevents them from being eroded into secondary microplastics upon degradation. This, in turn, reduces the residence time of biodegradable–compostable polymers compared to carbon–carbon backbone polymers, which leads to a reduction in the accumulation of plastics/microplastics in different areas [82]. 

Furthermore, the compost produced from separately collected biowaste is often contaminated with microplastics [67] that stem from carbon–carbon backbone polymer impurities, which are erroneously disposed off alongside the generated biowaste [78]. The contamination of biowaste with microplastics is significantly reduced if larger volumes of biowaste are diverted to industrial composting facilities. Moreover, agricultural mulch films can support the reduction in the microplastics in soil, which are intended to biodegrade in less than two years (EN 17033) without causing an accumulation of plastic particles in soils. Biodegradable–compostable polymers can further serve as substitutes for carbon–carbon backbone polymers in cosmetic products, which are intentionally supplemented with microplastics in the form of fillers, emulsifying agents, and peeling particles. However, the biodegradability of such intentionally added microplastics must be ascertained in the targeted environments, such as marine, freshwater, and soil environments (ISO 17756, ISO 18830, ISO 19769, and ISO 22404), and comprehensive ecotoxicity testing must be carried out [83]. 

### 2.2. Biobased Polymers

Biodegradability–compostability is related to the end-of-life characteristics of plastics, irrespective of the monomer source used for their preparation [27,84]. However, the bio-value proposition has an enhanced impact on reducing the global carbon footprint, which operates on the principle of a circular economy. It has been estimated that by substituting 65.8% of the fossil-based polymers with bio-based polymers, GHG emissions would be reduced by 241–316 MT CO_2_e/year. The European Commission, Japan, Korea, and Thailand are currently implementing strategies for promoting bio-based plastics [11].

**Figure 3:**
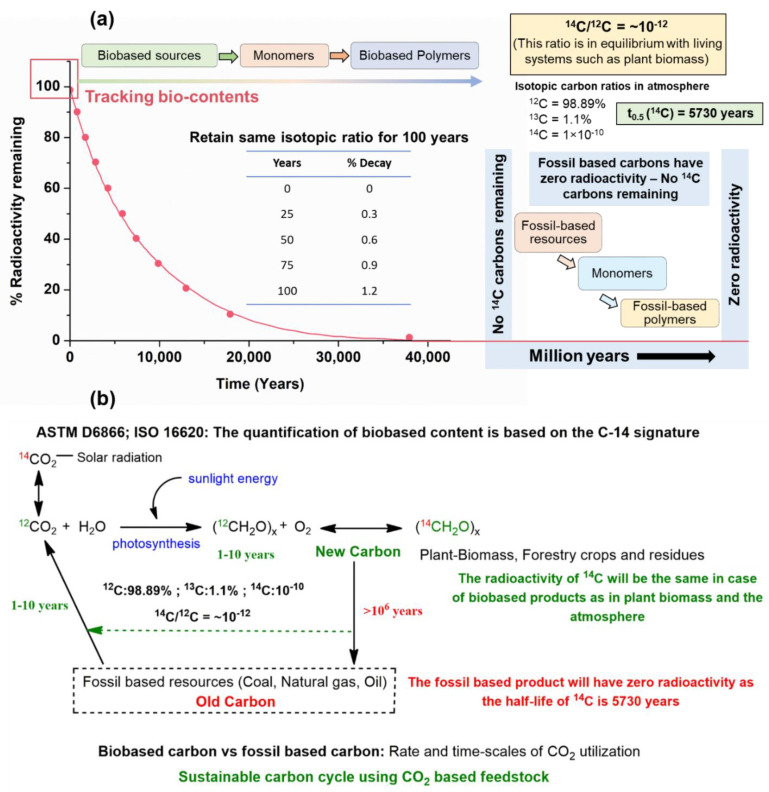
(**a**) Tracking the bio-content of polymers based on their C-14 signatures; (**b**) Illustration of the zero material carbon footprint using biobased sources. Reproduced with permission from [58].

Polymers that are fully biobased and biodegradable–compostable at their end-of-life stages (such as PLA, PHA, cellulose esters, and polysaccharides) may not be directly suitable for end-use applications due to their poor oxygen or water-barrier properties, [85], brittle nature, and/or thermomechanical stability [86,87]. Therefore, the current market strategy involves the combination of biobased and/or petroleum-based polymers, which are biodegradable–compostable at their end-of-life stages. Thus, the bio value proposition becomes significant from the environmental and commercial point of view. The carbon present in the environment in the form of CO_2_ can be incorporated into polymer backbone chains via photosynthesis on a time scale of 1–10 years when the fossil-based carbon in a polymer molecule is replaced with that of biobased carbon. Fossil-based plastics are formed over millions of years from plant biomass (Figure 3a), which cannot be credited with any removal of CO_2_ from the environment even over a time scale of one hundred years, which is the time frame used for the measurement of global warming potential (GWP100). The separation of natural carbon from fossil-based carbon thus becomes important in analyzing the bio-content in the commercialized polymer blends and composites that are gaining popularity. 

The biobased carbon content in polymers is determined independently via radiocarbon dating, which is used to identify the rate of ^14^C carbon decay via the most widely accepted method of accelerator mass spectrometry. Petrochemical feedstocks are formed over millions of years, thus negating the presence of the ^14^C isotope (with a half-life of 5730 years), which is otherwise present in plant biomass and the atmosphere [88]. The standard test methods that identify bio-based content are ASTM D6866 and ISO 16620 (Figure 3b), where the former relies on organic carbon content, while the latter accounts for organic and biogenic carbon [89]. The amount of CO_2_ removed from the environment by 1 kg of material can be calculated by determining a material’s biobased carbon content and applying stoichiometric calculations (for instance, 1 kg of PLA, which is 100% biobased, would remove 1.83 kg of CO_2_ from the environment). This carbon will be released back into the environment as CO_2_ at the end of life of the plastics; however, it is also captured by the next season’s biomass plantation, thereby resulting in a net-zero material carbon footprint. On the contrary, fossil-based polymers, such as PP or PE, would result in a net release of 3.14 kg CO_2_ into the environment for every 1 kg of PP or PE used [58]. Thus, the replacement of fossil-based carbon with biobased carbon would lead to a reduced carbon footprint and enable the technology to move towards a closed-loop ‘circular economy’. Nevertheless, the polymers that are derived from renewable sources and are biodegradable–compostable at their end of life will have a reduced environmental impact throughout their life cycle as compared to their exclusively biobased or biodegradable–compostable counterparts.

## 3. Outlook

Carbon–carbon backbone polymers were designed with little consideration for their ultimate disposal or the impact of the feedstocks that were used for their production. This approach has resulted in negative ecological impacts of such materials at their end-of-life stages. Plastics are long-lasting materials that have served myriad human needs; however, their key attribute of durability poses a serious threat to the environment when they enter waste streams. Further, their virtually indestructible character has significantly affected marine ecosystems and aquatic species. The replacement of carbon–carbon backbone polymers with those of biodegradable–compostable plastics in single-use applications constitutes a means of reducing the accumulation of waste in the environment. The world is evolving away from a linear economy towards a circular economy through redesigning carbon–carbon backbone polymers such that they are biodegradable–compostable at their end-of-life stages. Redesigning carbon–carbon backbone polymers by incorporating an ester backbone may be more suitable for biological processing in managed systems such as industrial composting. Such new polymers must be tested with respect to the international standards (ASTM/ISO) in order to examine their performance in composting facilities. Efforts may further be exerted toward designing compost systems that can replicate real-time environments for improved accuracy. Furthermore, a strong value-proposition may be realized by replacing fossil-based carbon with bio-based carbon to reduce the global environmental impact.

## 4. Conclusions/Future Perspectives

The strategy of re-designing carbon-carbon backbone polymers to labile ester backbone polymers, serves as an environmentally responsible solution for reducing the accumulation of plastic waste on land and oceans. Further, biodegradability is not only an inherent property of plastics, but also depends on the receiving environment. Therefore, plastics labelled as biodegradable-compostable must be diverted through specific systems for which they are certified as biodegradable. It becomes essential to recognize the key properties of plastics as well as receiving environments, while assessing the biodegradation of plastics, as per the ASTM/ISO standards. Further, replacing petro-fossil based carbon by plant-biomass carbon provides a strong bio-value proposition to reduce the carbon footprint and design polymers with a responsible end-of-life. Although standard protocols and test methods have been developed to test the biodegradability of plastics in receiving environments under standard laboratory conditions, it is essential to determine their degradability under real in situ environmental conditions to assure actual biodegradability. While biodegradable-compostable plastics do not alleviate all the problems associated with the plastic waste, they clearly provide for a responsible end-of-life, and can replace carbon-carbon backbone polymers in single-use applications.

## Figures and Tables

**Table 1:** Commercially available biodegradable–compostable polymers.

Source	Polymers	Commercial Name	Company	Ref.
**Biobased**	Polyhydroxyalkanoates (PHAs)	Nodax^®^	Danimer Scientific	[34]
		AmBio	Shenzhen Ecomann Biotechnology Co., Ltd.	[35]
		AONILEX^®^	Kaneka Corporation	[36,37]
		Solon^TM^	RWDC Industries	[38]
		Enmat	TianAn Biologic Materials Co., Ltd.	[39]
		AirCarbon	Newlight Technologies LLC	[40]
	Polylactic acid (PLA)	Luminy^®^ PLA	TotalEnergies Corbion	[41]
		NatureWorks^®^ PLA	NatureWorks LLC	[42]
	Polyurethane (PU)	Susterra^®^	Covation Biomaterials LLC	[43]
	Cellulosic polymers	NatureFlex^TM^	Futamura Chemical Co., Ltd.	[44]
**Fossil-based**	Polybutylene adipate-co-terephthalate (PBAT)	Ecoflex^®^	BASF	[45]
		Ecopond^®^	Kingfa	[46]
		Ecoworld^®^	Jinhui Zhaolong High Technology Co., Ltd.	[47]
		Tunhe PBAT	Xinjiang Blue Ridge Tunhe Sci.&Tech. Co., Ltd.	[48]
	Poly(ε-caprolactone) (PCL)	CAPA^®^	Ingevity^TM^	[49]
**Blends of fossil based and bio-based**	PBAT/PLA	Ecovio^®^	BASF	[50]
	PCL/Starch	Mater Bi	Novamont	[51]
	PLA/PBS	BioFlex® S 5630	FKuR Kunststoff GmbH	[52]
	Poly(trimethylene terephthalate) (PTT)	Sorona^®^	Covation Biomaterials^TM^	[53]
	Polybutyelene succinate (PBS)	BioPBS^TM^	PTT MCC Biochem Co., Ltd.	[54]
		Biobased Succinic Acid^®^	Succinity GmbH (Joint venture between Corbion and BASF)	[55]
		Biosuccinium^TM^	Reverdia	[56,57]

## Data Availability

Data sharing not applicable.
